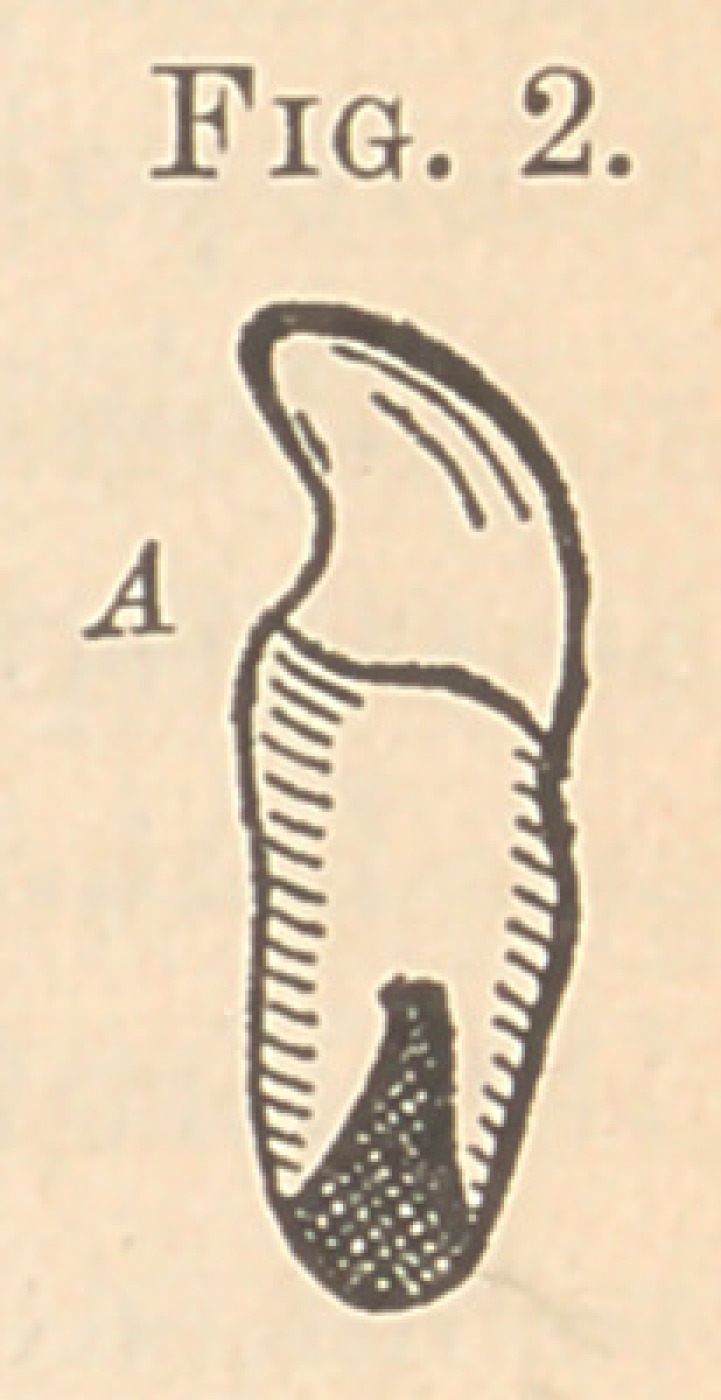# An Abnormal Position of a Left Central

**Published:** 1893-09

**Authors:** W. Irving Thayer

**Affiliations:** Williamsburgh, Mass.


### AN ABNORMAL POSITION OF A LEFT CENTRAL.

BY W. IRVING THAYER, M.D., D.D.S., WILLIAMSBURGH, MASS.

EARLY in July, 1893, while paying a visit to a well-known dentist in New York, I was invited into his operating-room in consultation upon a mal-positioned left central.

The lady, Mrs. M., aged about forty-five, had been the patient of E. Parmly Brown, D.D.S., from girlhood to that time. I found the left central had from some cause turned end for end, so that the crown pointed upward and was quite visible in the left nostril.

It will be seen by reference to the cuts that Fig. 2, which is a left lateral view, shows the crown to have a squatty appearance, a hump-back look, showing, in the opinion of the writer, a *double compression* in its formative or embryonic life that first curved the root at a spot in this tooth having the least resistance, and when calcareous deposits had gathered in greater quantity in the alveolar tissue to support the apical end, then the crown must needs "hump-back" itself, being largely composed of soft solids because it had not at this time received a normal supply of the necessary calcareous salts.

FIG. 1. FIG. 2.

The lateral plain view shows—Fig. 2—the tooth to be a little shorter in outside lines than in the labial aspect.

In Fig. 1 the curve was to the left. At *A*, Fig. 2, it will be observed that the major portion of the palatine surface is disintegrated with a dry, absorbed condition of the soft solids, seemingly leaving the lime salts in position, described as dry rot.

It is important to state that the right central and lateral and left lateral were erupted in normal position. Again, there had never been any appearance of supernumerary teeth.

Where did the crowding come from?

The tooth was extracted from the left nostril by Dr. Brown, *without the least pain* to Mrs. M., by the topical application of anæstheto-obtundent number two. It was a mere accident that the writer was present at the time. The lady had for many years desired to have the annoying tooth removed, but would not consent to take chloroform, ether, or nitrous oxide. She finally consented to the use of this anæsthetic after being assured that with it the operation could be made painless.

There are many interesting and instructive points in this case; but who is wise enough to inform the reader why this tooth erupted into the left nostril? What force sent it skyward? What influences were at work to make a fish-hook out of the root and a hump-back of the crown?

Mrs. M. had a cleft palate, so that the writer, who applied number two, made four swabs so curved that he could reach in back of the opening, upward and forward, and obtund in this direction, as well as more direct applications to all portions of the anterior nares and gums.

When a certain able gentleman saw the original tooth, and was informed of the "cleft," he said, "Oh, that tells the story of the end-for-end position of the tooth as well as its other crooked conditions. There was no room in front for this tooth to come down naturally, and it must needs go somewhere, so it developed upward. Cleft-palate cases are narrowed arches."

It is a good thing to be wise, but for some reason nature failed to contract Mrs. M.'s upper maxillary.

But what turned that left central completely around? This Dr. Brown or the writer failed to discover. It was not for lack of room, nor a gradual contraction of the arch as age advanced, for the "cleft" did not reach quite up to the rugæ, so that there was vault enough to prevent a doubling-up of the centre at median line.

A lower acute-angled alveolar forceps was used in extraction. There was a large flow of blood, and much joy expressed by the patient to be relieved of her burden in so satisfactory a manner.

The writer is unable to find any other case recorded of the removal of a tooth through the nostril. He knows of a root being extracted from a cadaver, but not from a patient. The upper lip was perfectly formed. The anæsthesia was so complete that a tumor could have been removed in addition to that of the tooth. There was much previous local inflammation, which was stimulated to such an extent by the anæsthetic that the parts took on almost immediately a healthy condition.

## Figures and Tables

**Fig. 1. f1:**
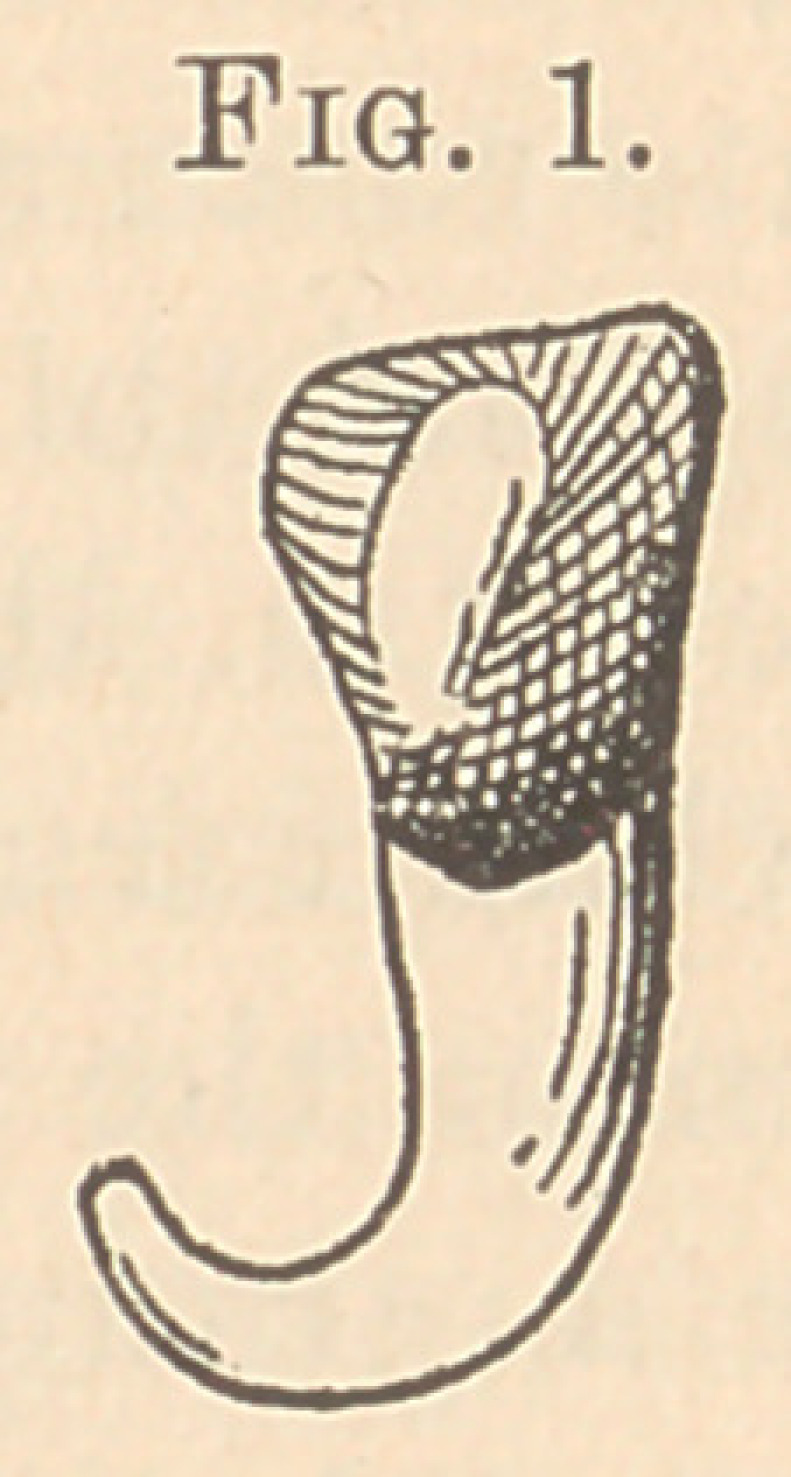


**Fig. 2. f2:**